# Purple Urine Bag Syndrome May Not Be Benign: A Case Report and Brief Review of the Literature

**DOI:** 10.1155/2013/863853

**Published:** 2013-06-20

**Authors:** Mukul Bhattarai, Hamid Bin Mukhtar, Thomas Walter Davis, Alok Silodia, Hitekshya Nepal

**Affiliations:** ^1^Department of Internal Medicine, Geisinger Medical Center, 100 North Academy Avenue, Danville, PA 17822, USA; ^2^Woodside Family Health Center, 5718 Woodside Ave, NY 11377, USA

## Abstract

Purple urine bag syndrome (PUBS) is a rare condition in which there is purple discoloration of the urine with its collecting bag and associated tubing occurs. It is considered a benign condition. We report an unusual case of PUBS in an 87-year-old female from nursing home who had a history of recurrent UTI. She also had a history of ureteral obstruction requiring left nephrostomy tube. She was brought to emergency department with altered mental status which developed five days after the occurrence of purple discoloration of the urinary bag. Her urine culture grew vancomycin-resistant *Enterococci* (VRE) and *Pseudomonas aeruginosa*. She died within three days of hospitalization despite intensive care in tertiary center. This case highlights that PUBS may not always be benign and should be approached on a case-by-case basis because it may signal the underlying UTI which might be very difficult to treat. Failure of recognition of this peculiar color early could delay the appropriate intervention leading to fatal complication. This case also represents the rare occurrence of PUBS in the setting of nephrostomy tube.

## 1. Introduction

The reason of occurrence of PUBS is still unknown [1]. However, there are several predisposing factors commonly implicated with PUBS [2, 3]. It occurs in the setting of chronic UTI due to several pathogens producing enzymes which make the urine and its containing bag and tube purple. However, it is considered benign [2, 3]. We present a case of PUBS as a manifestation of complicated UTI. 

## 2. Case Description

An 87-year-old Caucasian female who had a medical history of dementia, hypertension, hyperlipidemia, and recurrent UTI presented to our tertiary care center with altered mental status for one day. She had a history of bilateral ureteral obstruction which required left nephrostomy tube and right ureteral stent 3 years ago. She also had end-stage renal disease (ESRD) and was dependent on hemodialysis for the past 2 years. Her right ureteral stent and left nephrostomy tube were changed by her urologist one month prior to this admission. She was a nursing home resident and had poor baseline functional activity. Her daily medication included aspirin 81 mg, metoprolol 50 mg, and simvastatin 40 mg. She was also on several bowel regimens like senna and colace for her chronic constipation. There was no recent change in her bowel movement reported. However, her last bowel movement was three days ago. 

She was receiving regular hemodialysis three days in a week. 

Her history was also notable for multiple hospitalizations due to chronic and recurrent UTI in the past ten years due to several bacteria, namely, *E. coli*, *Pseudomonas aeruginosa*, *Klebsiella pneumonia*, *Enterobacter cloacae*, MRSA (methicillin-resistant* Staphylococcus aureus*), *Acinetobacter baumannii*, *Providencia rettgeri*, *Enterococcus faecium*, *Proteus mirabilis*, and *Morganella morganii*. 

She had recent UTI two months ago due to *E. coli* and *Morganella morganii* which was successfully treated with ciprofloxacin. 

**Figure 1:**
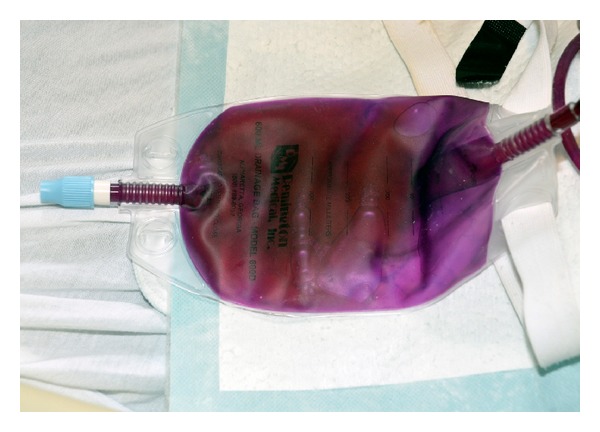
Dark purple discoloration of urinary bag.

**Figure 2:**
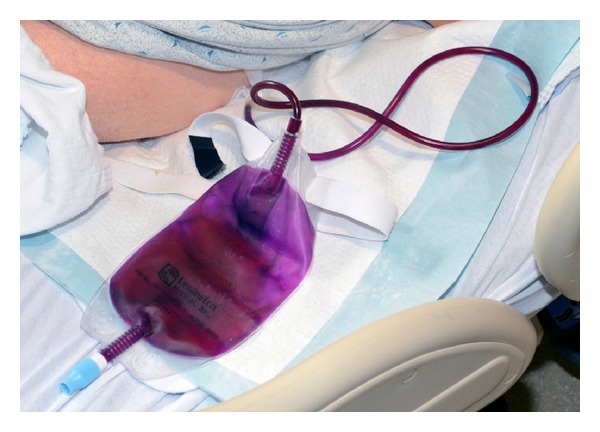
Dark purple discoloration of entire nephrostomy tube and urinary bag.

At this time on presentation, she was afebrile. Her blood pressure was 84/32 mmHg, respiratory rate 25 per minute, and pulse rate 85 beats per minute. On physical examination, she was not in distress though she appeared confused, not orienting to time and place. There was no focal deficit on examination. No neck rigidity was appreciated. But her exam was remarkable for finding a dark purple discoloration of the entire left sided nephrostomy tube and urinary bag which was suggestive of PUBS (Figures 1 and 2). Upon further inquiry, her nursing home reported that discoloration of her urinary bag and tube started five days prior to development of altered mental status and was thought to be benign because it was not causing any symptoms to the patient. However, in the past 2 days, she was gradually feeling weak and later became confused which eventually brought her to the hospital. 

Laboratory workup showed hemoglobin of 10 gm/dL which was at her baseline, and total leukocyte count was at 18,000/microliter (normal 4000–12,000/microliter) with 8 percent bands. Computed tomography (CT) head did not reveal any acute cause of her confusion. Urine analysis showed specific gravity of  1.013, alkaline urine of pH 7.5, and large amount of esterase and nitrates. There were numerous bacteria and white count reported in urine analysis. She started empiric antibiotic treatment with vancomycin and cefepime. Her urine culture grew *Enterococci* species more than 105,000 colony-forming units (CFU) per milliliter of urine which was resistant to cefepime, ampicillin, vancomycin, nitrofurantoin, streptomycin, and gentamycin. The culture also grew *Pseudomonas aeruginosa* 1700 colonies/mL. Her clinical status gradually deteriorated, and she died on the third day of hospitalization despite aggressive management in ICU (intensive care unit). 

## 3. Discussion

PUBS was first described in the literature in 1978 [4]. It is considered a rare phenomenon [5]. However, several small studies report the prevalence of PUBS as 8.3%–16.7% in patients with long-term urinary catheterization [2, 3, 6]. It is common in female gender and is associated with alkaline urine, constipation, dementia, chronic UTI, institutionalization, and the use of a plastic urinary catheter [2, 3, 5–7]. It might also occur in an ESRD patient who is on hemodialysis [8]. Our patient also had all typical risk factors of PUBS: elderly female, dementia, nursing home resident, chronic constipation, history of chronic and recurrent UTI, hemodialysis dependent for ESRD, and presence of alkaline urine. Typically PUBS is seen in patients with a chronic Foley's catheter in the bladder. 

There are only few cases reported in the literature of PUBS with underlying nephrostomy [1, 9, 10]. Our patient is also a rare case of purple urine bag syndrome in the setting of nephrostomy tube.

There are several bacterial species associated with the purple discoloration of urine and its container such as *Providencia* species, *E. coli*, *Proteus* species, *Pseudomonas* species, *Klebsiella pneumoniae*, *Morganella* species, and *Enterococcus* species. It is less commonly caused by *Citrobacter* species, *Staphylococcus* species, *Streptococcus* species, and even MRSA [2, 11, 12]. Our patient had chronic and recurrent history of UTI due to many types of these pathogens. Prior to her death, vancomycin-resistant *Enterococcus* and *Pseudomonas aeruginosa* were isolated from her urine culture. 

**Figure 3:**
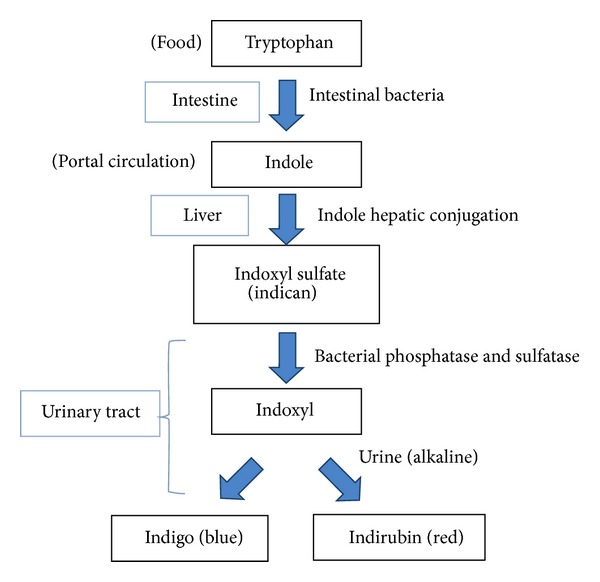
Pathogenesis of PUBS (with courtesy of Hadano et al. [3]).

The pathogenesis of PUBS starts from metabolism of tryptophan by intestinal bacteria finally leading to the formation of pigments (indigo and indirubin) in the urine in the presence of bacterial phosphatase and sulfatase. The combination of indigo and indirubin causes the purple discoloration of urine as depicted in Figure 3 [3]. Moreover, higher bacterial load in the urine is considered an important predisposing factor [12]. Chronic constipation as in our case also promotes bacterial overgrowth thereby enhancing degradation of dietary tryptophan [13]. 

The several reports including the recently published updated article in 2012 by Hadano et al. focus on the benign nature of PUBS because it almost always appears asymptomatic, harmless and not requiring intensive treatment [3]. However, the treatment of underlying UTI, control of constipation, appropriate nutritional management, and proper urologic sanitation are essential for its treatment [5, 14]. 

But there are few case reports of the alarming nature of PUBS including ours, requiring aggressive treatment including intravenous antibiotic. Pillai et al. reports a 68-year-old female who died on the same day of developing UTI with PUBS [11]. In another report of PUBS, UTI was treated with intravenous antibiotic and catheter exchange to nonteflon type resulting in complete resolution of UTI and purple discoloration of urine [5]. Similarly, there was a case report of an 81-year-old man who presented with purple discoloration of urine to the emergency department and was successfully treated with a course of cefuroxime [13]. Tasi et al. also reported the two cases of PUBS in immunocompromised patients which showed progression to Fournier's gangrene [7]. Hence, PUBS is not always benign. Early recognition and intervention are warranted in certain cases such as symptomatic PUBS, immunocompromised patients, history of recurrent UTIs, institutionalized patients, and also in those who are not having a proper care of the urinary catheters and proper sanitation. 

At present, there is no consensus in the management of asymptomatic PUBS which is often overlooked. Failure of early recognition of this peculiar color could delay the appropriate intervention leading to fatal complication as in our case. Our case also demonstrated the necessity for the formulation of the standard practice guidelines to treat asymptomatic PUBS. Therefore, further research and large scale studies would be beneficial in asymptomatic PUBS to determine the high risk factors which could potentially lead to severe complication. 

## 4. Conclusion

Though PUBS is usually considered benign, we highlight that an approach to the management of PUBS should be dealt on a case-by-case basis. However, we need to be more cautious on this unique phenomenon in those patients who already have a history of chronic and recurrent UTI caused by several pathogens because there is a possibility of multidrug-resistant organisms causing PUBS making it more difficult to be treated. In those cases, early recognition of purple discoloration should not be overlooked to prevent the severe complication. Large scale studies would be beneficial to recognize the high risk patients in order to intervene early and appropriately.
